# Phloretin Prevents Diabetic Cardiomyopathy by Dissociating Keap1/Nrf2 Complex and Inhibiting Oxidative Stress

**DOI:** 10.3389/fendo.2018.00774

**Published:** 2018-12-20

**Authors:** Yin Ying, Jiye Jin, Li Ye, Pingping Sun, Hui Wang, Xiaodong Wang

**Affiliations:** ^1^Department of Pharmacy, Tongde Hospital of Zhejiang Province, Hangzhou, China; ^2^Department of Rehabilitation, Tongde Hospital of Zhejiang Province, Hangzhou, China; ^3^Department of Nursing, Tongde Hospital of Zhejiang Province, Hangzhou, China; ^4^Department of Vascular Surgery, Tongde Hospital of Zhejiang Province, Hangzhou, China

**Keywords:** phloretin, Nrf2, Keap1, diabetic cardiomyopathy, oxidative stress

## Abstract

Hyperglycemia induces chronic inflammation and oxidative stress in cardiomyocyte, which are the main pathological changes of diabetic cardiomyopathy (DCM). Treatment aimed at these processes may be beneficial in DCM. Phloretin (PHL), a promising natural product, has many pharmacological activities, such as anti-inflammatory, anticancer, and anti-oxidative function. The aim of this study was to investigate whether PHL could ameliorate the high glucose-mediated oxidation, hypertrophy, and fibrosis in H9c2 cells and attenuate the inflammation- and oxidation-mediated cardiac injury. In this study, PHL induced significantly inhibitory effect on the expression of pro-inflammatory, hypertrophy, pro-oxidant, and fibrosis cytokines in high glucose-stimulated cardiac H9c2 cells. Furthermore, PHL decreased the levels of serum lactate dehydrogenase, aspartate aminotransferase, and creatine kinase-MB, and attenuated the progress in the fibrosis, oxidative stress, and pathological parameters via Kelch-like ECH-associated protein 1 (Keap1)/nuclear factor E2-related factor 2 (Nrf2) pathway in diabetic mice. In additional, molecular modeling and immunoblotting results confirmed that PHL might obstruct the interaction between Nrf2 and Keap1 through direct binding Keap1, and promoting Nrf2 expression. These results provided evidence that PHL could suppress high glucose-induced cardiomyocyte oxidation and fibrosis injury, and that targeting Keap1/Nrf2 may provide a novel therapeutic strategy for human DCM in the future.

## Introduction

Diabetes mellitus (DM) is an emerging global health problem. Diabetic cardiomyopathy (DCM) is the one of the major complications of diabetes that causes mortality and morbidity in diabetic patients (1). DCM starts with diastolic dysfunction in patients with type-1 (T1DM) or type-2 (T2DM). Previous studies showed that DCM presented with structural and functional abnormalities of the myocardium, leading to increased risks for myocardial fibrosis, ventricular hypertrophy, and heart failure (2, 3). There are several physiological mechanisms related to the pathogenesis for DCM, including insulin resistance signaling, cardiac inflammation, oxidative stress, endoplasmic reticulum stress, etc. (4). Increasingly, evidence demonstrated that oxidative stress may be the most common feature linking diabetes-induced alterations to the development of cardiac dysfunction.

The Keap1/Nrf2 pathway is the most crucial anti-oxidative mechanism that protects cells from oxidative stress (5). Nrf2 is a master regulator of redox status and cellular detoxification responses by inducing the expression of multiple downstream anti-oxidant genes, including heme oxygenase (HO-1), nicotinamide adenine dinucleotide phosphate-H (NADPH), quinineoxidoreductase-1 (NQO-1), and glutamate-cysteine ligase catalytic (GCLC) (6). Recently, it was reported that Nrf2 prevented T2DM-induced cardiac injury (7). Furthermore, another study indicated that fibroblast growth factor-21 (FGF21) prevented diabetic cardiomyopathy via AMPK-AKT2-Nrf2 mediated anti-oxidation and lipid-lowering effects in the heart (8). Therefore, controlling cytoprotective oxidative stress response enzymes in DCM via Keap1/Nrf2 pathway targeting is a potential important strategy.

**Figure 1:**
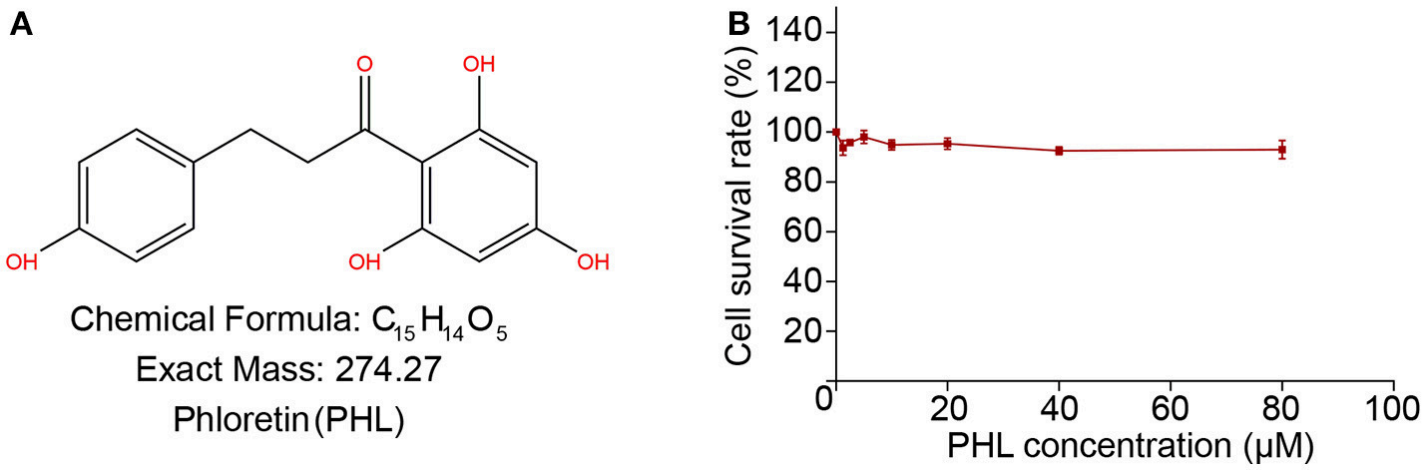
The chemical structure of Phloretin **(A)**. MTT assay tests the effect of PHL on H9c2 survival rate after 24 h treatment **(B)**.

Phloretin (PHL, Figure 1A) is a dihydrochalcone flavonoid found in peel and root skin of frequently consumed vegetables and fruits. PHL showed numerous biological and pharmacological activities, such as anti-inflammatory, antioxidant, and anti-cancer in various disease models (9–12). Previously, our group found that PHL significantly inhibited tert-Butyl hydroperoxide (TBHP) induced oxidation. However, the potential of PHL for the treatment of DCM and the molecular mechanisms underlying the actions of PHL remain unclear. In this study, we evaluated the protective effect of PHL in hyperglycemia-induced oxidative stress and cardiac injury *in vitro* and *in vivo*. Subsequently, molecular modeling and immunoblotting were used to explore the underlying mechanisms and possible targets of PHL. Our results revealed that PHL could prevent cardiac injury in T1DM by attenuating hyperglycemia-induced oxidative stress and hypertrophy. We identified Keap1, a negative regulator of Nrf2, as the possible target for PHL. Overall, our study provided crucial evidence that PHL may be a novel therapeutic agent for the treatment of DCM.

## Materials and Methods

### Reagents, Cell Culture and Treatment

Phloretin (purity 98%, verified by high-performance liquid chromatography; molecular weight = 274.27) was purchased from Aladdin (Shanghai, China). Phloretin was dissolved in DMSO for *in vitro* experiments and in CMC-Na (0.5%) for *in vivo* experiments, both stored at 4°C for further use. H9c2 embryonic rat heart-derived cell line was purchased from the Shanghai Institute of Biochemistry and Cell Biology (Shanghai, China) and cultured in DMEM medium (Gibco, Eggenstein, Germany) including 5.5 mmol/l of D-glucose supplemented with 10% FBS, 100 U/ml of penicillin, and 100 mg/ml of streptomycin. For the high glucose-treated group (HG), cells were incubated with a DMEM medium, which was contained 33 mmol/L of glucose. Glucose and streptozotocin (STZ) were obtained from Sigma-Aldrich (St. Louis, MO). Haematoxylin-eosin (H&E) was purchased from Beyotime (Nantong, China). Masson's trichrome kits was obtained in Solarbio (Beijing, China).

Antibodies for Nrf2 (#12721), TGF-β (#3711), Keap1 (#8047), GAPDH (#5174), and secondary antibodies (mouse #7076, rat #7077) were obtained from Cell Signaling Technology (Danvers, USA). RIPA lysis buffer was purchased from Boster Biological technology (Wuhan, China).

### Animals and Treatment

Male C57BL/6 mice weighing 20–22 g were obtained from Zhejiang Animal Center (Hangzhou, China). The mice were housed at a constant room temperature with a 12:12 h light-dark cycle and fed with a standard rodent diet and water. All animal experimental procedures complied with the “The Detailed Rules and Regulations of Medical Animal Experiments Administration and Implementation” (Document No. 1998–55, Ministry of Public Health, PR China), and were approved by the Tongde Hospital of Zhejiang Province Animal Policy and Welfare Committee (Approval Document No. SCXK2014-0001).

Eighteen mice were randomly divided into three groups. Twelve mice were received intraperitoneal (i.p.) injection of STZ at the dose of 100 mg/kg formulated in 100 mM citrate buffer (pH 4.5) for 1 time, blood glucose levels were detected using a glucometer, control animals received buffered saline only. Six mice treated with phloretin at 10 mg/kg through i.g. after injection STZ 8 days. At Day 56 after STZ induction, the mice were killed under anesthesia, and then blood samples were collected. At the time of death, the heart tissues were removed.

### Determination of Serum Aspartate Aminotransferase (AST), Lactate Dehydrogenase (LDH) and Creatine Kinase (CK-MB), Malondialdehyde (MDA), and Superoxide Dismutase (SOD)

Serum levels of AST, LDH, and CK-MB and supernatant levels of MDA and SOD were analyzed by commercial ELISA kits refer to the manufacturers' instruction (Nanjing Jiancheng Bioengineering Institute, Nanjing, China).

### Heart Histopathology

Heart tissue was fixed in 10% formalin for 24 h, embedded in paraffin, and sectioned at 5 μm. Then, the heart sections were deparaffinized, rehydrated, and stained with hematoxylin and eosin (H&E). Cardiac fibrosis was tested by Masson's trichrome staining for collagen deposition as described previously. To estimate the extent of damage, the specimen was observed under a light microscope (Nikon, Japan).

### Cell Cytotoxicity

Before PHL treatment, seeding cells into 96-well plates with 5,000 cells/well. Adding PHL into wells with various doses and incubated for 24 h. After treatment, MTT was added to each well (1 mg/ml), incubated at 37°C for 4 h. The formazan crystal was dissolved with DMSO, 150 μL/well. The absorbance was detected at 490 nm on a microplate reader. Cell cytotoxicity was expressed as the percentage of MTT reduction compared to control.

### Rhodamine Phalloidine Staining

For hypertrophy, cells were fixed with 4% paraformaldehyde, permeabilized with 0.1% Triton X-100, and stained with rhodamine phalloidin at a concentration of 50 μg/mL for 30 min. Nuclei were stained with the DAPI at room temperature for 5 min. Immunofluorescence was viewed and captured using Nikon fluorescence microscope (Nikon, Japan).

### RNA Isolation and Real-Time PCR (q-PCR)

Total RNA was extracted from the heart tissues and cells by using Trizol reagent (Invitrogen, Carlsbad, CA) according to each manufacturer's protocol. Both reverse transcription and quantitative PCR were carried out using a two-step M-MLV Platinum SYBR Green qPCR SuperMix-UDG kit (Invitrogen, Carlsbad, CA). Eppendorf Mastercycler ep realplex detection system (Eppendorf, Hamburg, Germany) was applied to q-PCR analysis. The primers of genes including NQO-1, HO-1, Nrf2, GCLC, CTGF, collagen-1, TGF-β, ANP, BNP, β-MyHC, and β-actin were obtained from Invitrogen (Shanghai, China). The primer sequences were listed in Table S1. Comparative cycle time (Ct) was used to determine fold differences between samples and normalized to β-actin.

### Western Blot Analysis

Heart tissues and harvested cell pellets were homogenized in RIPA lysis buffer (Santa Cruz Biotechnology, Santa Cruz, CA) to obtain total protein or nuclear protein extracted using a nuclei isolation kit (NUC201, Sigma-Aldrich). Western blot assay was performed for target protein quantification, as described previously. The proteins were separated by 10% sodium dodecyl sulfate (SDS)-polyacrylamide gel electrophoresis (PAGE) and transferred to a nitrocellulose membrane. Membranes were blocked with 5% non-fat milk for 1 h and incubated overnight at 4°C with the specific antibodies over-night in 4°C. Immunoreactive bands were detected by incubating with secondary antibody conjugated with horseradish peroxidase and visualizing using enhanced chemiluminescence reagents (Bio-Rad, Hercules, CA). The amounts of the proteins were analyzed using Image J analysis software and normalized to GAPDH.

### Co-immunoprecipitation (Co-IP) Analysis

Nrf2 was co-precipitated with Keap1 from cardiac tissues to detect the association of Nrf2 with Keap1. Cardiac tissue extracts (500 μg) were incubated with anti-Nrf2 antibody (0.5 μg) at 4°C overnight and then precipitated with protein A agarose (Beyotime, Shanghai, China) for 3 h. The Keap1/Nrf2 complex level in the beads was further detected by western blot.

### Intracellular ROS Measurement

The ROS production was measured by using the ROS-sensitive dye, 2,7-dichlorodihydrofluorescein diacetate (DCFH-DA, Beyotime Biotechnology, China) as an indicator, as described previously. To measure ROS production in tissues and cell, dihydroethidium (DHE) staining was performed as described previously (13).

### Construction of the Initial Structure of the Keap1/PHL Complex

The atomic co-ordinates of mouse Keap1 was download from the Protein Data Bank (PDB code: 5CGJ) (14). Next, the structure of Keap1 was preprocessed by VMD software (15). The AutoDock 4.2.6 package were used to predict the possible binding pose between Keap1 and PHL (16). Before molecular docking, the Keap1 and PHL were prepared by AutoDockTools 1.5.6 package, including adding missing hydrogen atoms and Gasteiger partial charges. After that, a grid box size of 22.5 Å × 22.5 Å × 22.5 Å was assigned, which covered almost the entire binding site of Keap1. During molecular docking, trials of 100 dockings, Lamarckian Genetic Algorithm (LGA) was used to globe conformational sampling, and other parameters were set as default. The lowest predicted binding energy conformation was used to further molecular dynamics (MD) simulation analysis.

### Conventional Molecular Dynamics (MD) Simulation and Gaussian Accelerated Molecular Dynamics (GaMD) Simulation

The structural optimization of PHL was conducted using B3LYP combined with 6 – 31+G^*^ basis set and RESP fitting method was applied for charge derivation based on the optimal conformation. The ff14SB force field was employed for the Keap1 protein and the general amber force field (gaff2) for the PHL (17, 18). The Keap1/PHL complex was solvated into TIP3P water box with boundary extended 12 Å away from any solute atom. The counter ions of Na^+^ were added to maintain the electroneutrality.

Before productive simulation, three consecutive minimization stages were applied to relax the system, including 5,000 steps of steepest descent and 5,000 steps of conjugate gradient steps. Firstly, energy minimization of only hydrogen atoms, followed by water molecules and counter ions, were performed with harmonic constraint potential of a 5.0 kcal mol^−1^ Å^−2^. Thereafter, the whole system was minimized without any constraint. The systems were gradually heated to 310 K in 100 ps, followed by 600 ps density equilibration. Finally, 200 ns conventional MD simulations and 400 ns GaMD simulation were performed in the NPT ensemble and NVT ensemble, respectively. In particular, the dual potential boost method was employed in the GaMD simulation (19, 20). The boost parameters were calculated from an initial ~4 ns NVT conventional MD simulation. During these simulations, the temperature were maintained by the Langevin temperature equilibration scheme (21). The long-range electrostatic interaction (cutoff = 10.0 Å) was used to evaluate direct space interaction with the particle-mesh Ewald (PME) method (22). All bonds involving hydrogen atoms were constrained by using the SHAKE algorithm (23). The simulation trajectories were processed by CPPTRAJ module in Amber 16 package (24). The Binding free energy decomposition was calculated by molecular mechanics/generalized Born surface area (MM/GBSA) method based on 200 snapshots extracted from the last 40 ns conventional MD simulation trajectory.

### Statistical Analysis

Statistical analysis was performed using Student's *t*-test, and ANOVA as appropriate, with Tukey or Bonferroni *post hoc*-tests. All data were analyzed with GraphPad Prism 5.0 (Graphpad Software, Inc.); *p* < 0.05 were considered significant.

## Results

### The Cytotoxicity of PHL

We investigated the cytotoxicity of PHL on H9c2 cells by MTT assay. As shown in Figure 1B, treatment of H9c2 cells with increasing concentrations of PHL (2.5, 5, 10, 20, 40, and 80 μM) for 24 h showed that even at 80 μM, PHL was relatively non-toxic to H9c2 cells. These results indicated that PHL had no obvious toxic effect on H9c2 cells. Finally, 10 μM PHL was selected for subsequent *in vitro* experiments.

### PHL Reduced Hyperglycemia-Induced ROS Levels Through Regulation of Nrf2 Antioxidant Responses in H9c2 Cells

**Figure 2:**
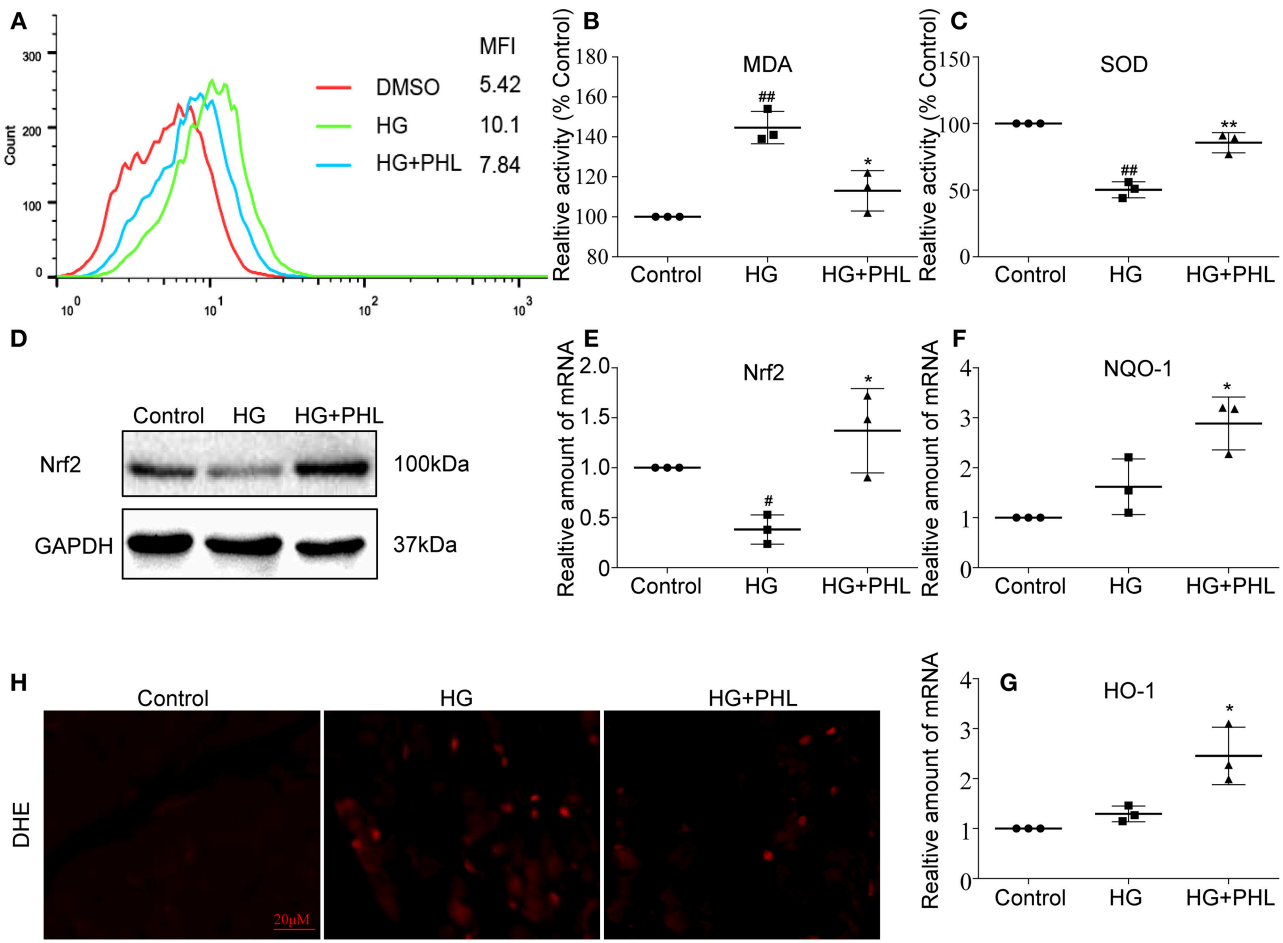
PHL reduced hyperglycemia-induced ROS levels through induction of Nrf2 anti-oxidant responses in H9c2 cells. **(A)** PHL inhibited high glucose-induced ROS generation. H9c2 (1 ^*^ 10^6^) cells pretreated with PHL (10 μM) for 1 h were incubated with HG (33 mM) for 3 h. DCFH-DA probes loaded and cells were processed by flow cytometry analysis for O_2_ level, and mean fluorescence intensity (MFI) value was determined. **(B,C)** H9c2 (5 ^*^ 10^5^) cells pretreated with PHL (10 μM) for 1 h and incubated with HG (33 mM) for 6 h. Levels of malondialdehyde (MDA) **(B)** in lysates prepared from H9c2 cells and enzymatic activity of superoxide dismutase (SOD) **(C)** as measured using colorimetric assays. **(D)** H9c2 (1 ^*^ 10^6^) cells were pre-treated with PHL (10 μM) for 1 h and then incubated with HG (33 mM) for 12 h. The cell lysates were immunoblotted for Nrf2, with GAPDH as a loading control. **(E–G)** Total RNAs were extracted and the mRNA levels of Nrf2, HO-1, and NQO-1 were detected by RT-qPCR. Cells were treated as in **(B)**. **(H)** Staining of cultured H9c2 cells with DHE. DHE generates red fluorescence product (ethidium) in the presence of ROS. Cells were treated as in **(A)**. Data are presented as mean ± SEM. ^*^P < 0.05, ^**^P < 0.01 vs. HG group; ^#^*P* < 0.05, ^##^*P* < 0.01 vs. Control group.

A large amount of evidence have shown that reactive oxygen species (ROS) play an important role in the pathogenesis of DCM (25). Therefore, we investigated the effect of PHL on hyperglycemia-induced oxidative stress. Firstly, we measured the effects of PHL on hyperglycemia-induced ROS generation and redox status markers. The hyperglycemia-induced group demonstrated markedly increased ROS generation, which significantly reduced by pre-treatment with PHL in hyperglycemia-induced H9c2 cells (Figure 2A). PHL also decreased the levels of MDA, a natural byproduct of lipid peroxidation (Figure 2B) and enhanced the enzyme SOD activity (Figure 2C). Dihydroethidium (DHE) reacts with superoxide anions to form a red fluorescent product, allowing signal visualization, and measurement of intracellular ROS in H9c2 cells. As shown in Figure 2H, PHL attenuated hyperglycemia-induced ROS production. These findings indicated that PHL is a potential inhibitor for hyperglycemia-induced ROS production.

As reported as Foresti et al. (26), hyperglycemia downregulated Nrf2 *in vitro* and *in vivo*. Nrf2 regulates the transcription of genes coding for anti-oxidant and detoxifying proteins, such as HO-1, NADPH, NQO-1, glutathione peroxidase-2, GCLC, and glutathione S-transferase. As shown in Figure 2D, the hyperglycemia-mediated downregulation of Nrf2 was prevented by the pre-treatment with PHL. Subsequently, we evaluated the expression levels of Nrf2, NQO-1, and HO-1 by pre-treating H9c2 cells with PHL for 30 min, followed by stimulation with high glucose (33 mM) for 24 h. The gene expression showed that Nrf2 (Figure 2E), NQO-1 (Figure 2F), and HO-1 (Figure 2G) were significantly increased in the PHL pre-treated group despite hyperglycemia induction. These results indicated that PHL inhibited the hyperglycemia-induced oxidation through Nrf2 gene in H9c2 cells.

### PHL Attenuated Hyperglycemia-Induced Cell Fibrosis and Hypertrophy in H9c2 Cells

**Figure 3:**
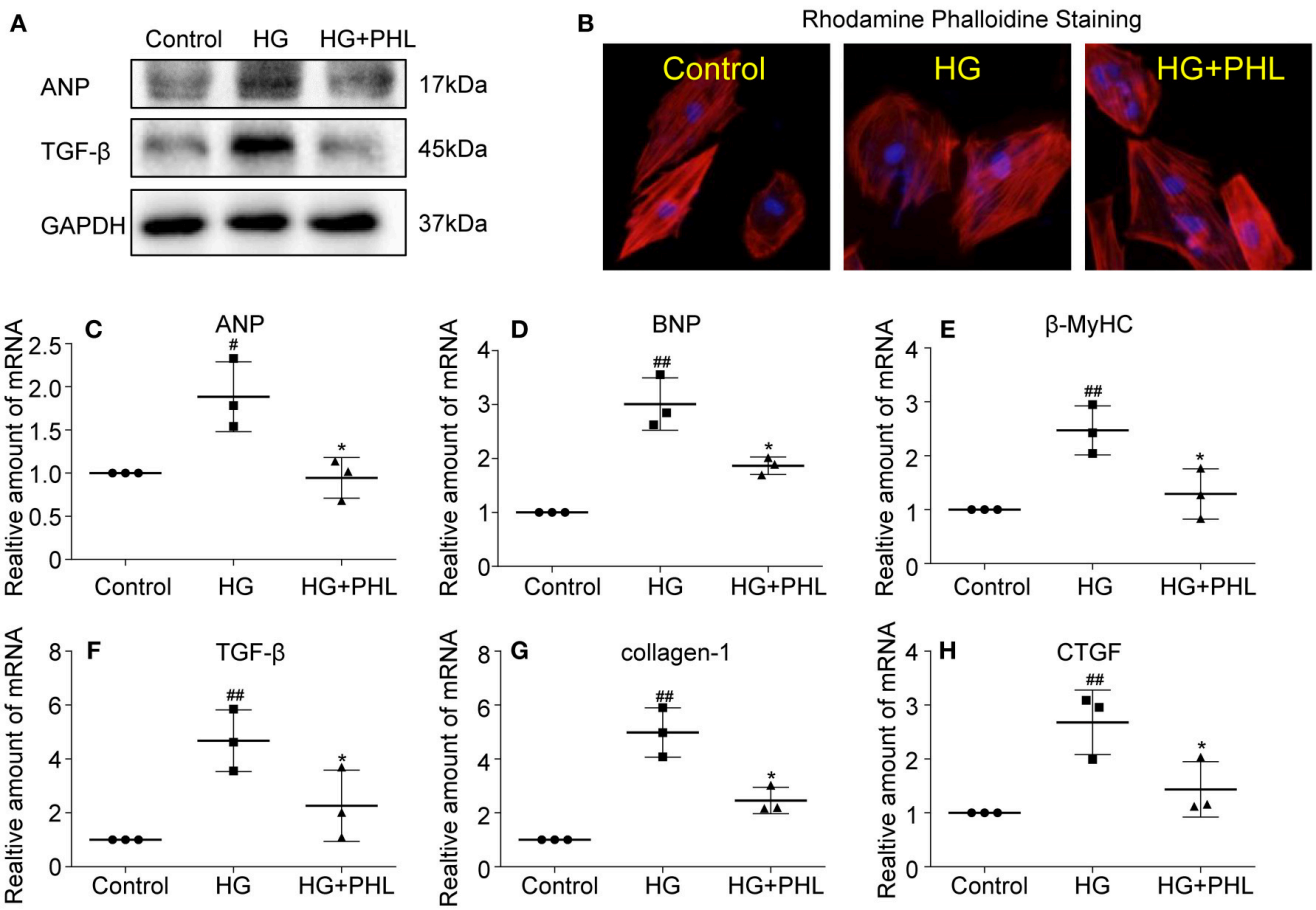
PHL reduced hyperglycemia-induced hypertrophy and fibrosis in H9c2 cells. H9c2 (1 ^*^ 10^6^) cells were pre-treated with PHL (10 μM) for 1 h and then incubated with HG (33 mM) for 24 h. **(A)** The cell lysates were immunoblotted for ANP, TGF-β, with GAPDH as a loading control. **(B)** The cell sizes were detected by Rhodamine Phalloidin/DAPI immunofluorescence staining. Total RNAs were extracted and the mRNA levels of ANP **(C)**, BNP **(D)**, β-MyHC **(E)**, TGF-β **(F)**, collagen-1 **(G)**, and CTGF **(H)** were detected by RT-qPCR. Data are presented as mean ± SEM. ^*^P < 0.05 < 0.01 vs. HG group; ^#^*P* < 0.05, ^##^*P* < 0.01 vs. Control group.

Myocardial fibrosis and hypertrophy are the major mechanisms contributing to cardiomyocyte remodeling in DCM (2, 27), so we assessed the effect of PHL on hyperglycemia-induced cardiac fibrosis and hypertrophy in H9c2 cells. As shown in Figure 3A, increased protein levels of hypertrophy marker atrial natriuretic peptide (ANP) and fibrosis marker TGF-β were observed in hyperglycemia-induced cells. These increases were significantly ablated by PHL pre-treatment. Meanwhile, RT-qPCR analysis revealed that PHL inhibited hyperglycemia-induced increases in hypertrophy factors, including ANP (Figure 3C), brain natriuretic peptide (BNP, Figure 3D) and β-myosin heavy chain (β-MyHC, Figure 3E) as well as fibrosis factors, such as transforming growth factor-β (TGF-β, Figure 3F), collagen-1 (Figure 3G), and connective tissue growth factor (CTGF, Figure 3H) gene expression. Rhodamine phalloidin staining also demonstrated an ablated hyperglycemia-induced cell size increase by PHL pre-treatment (Figure 3B).

### PHL Prevented Cardiomyocyte Injury in Diabetic Mice

**Figure 4:**
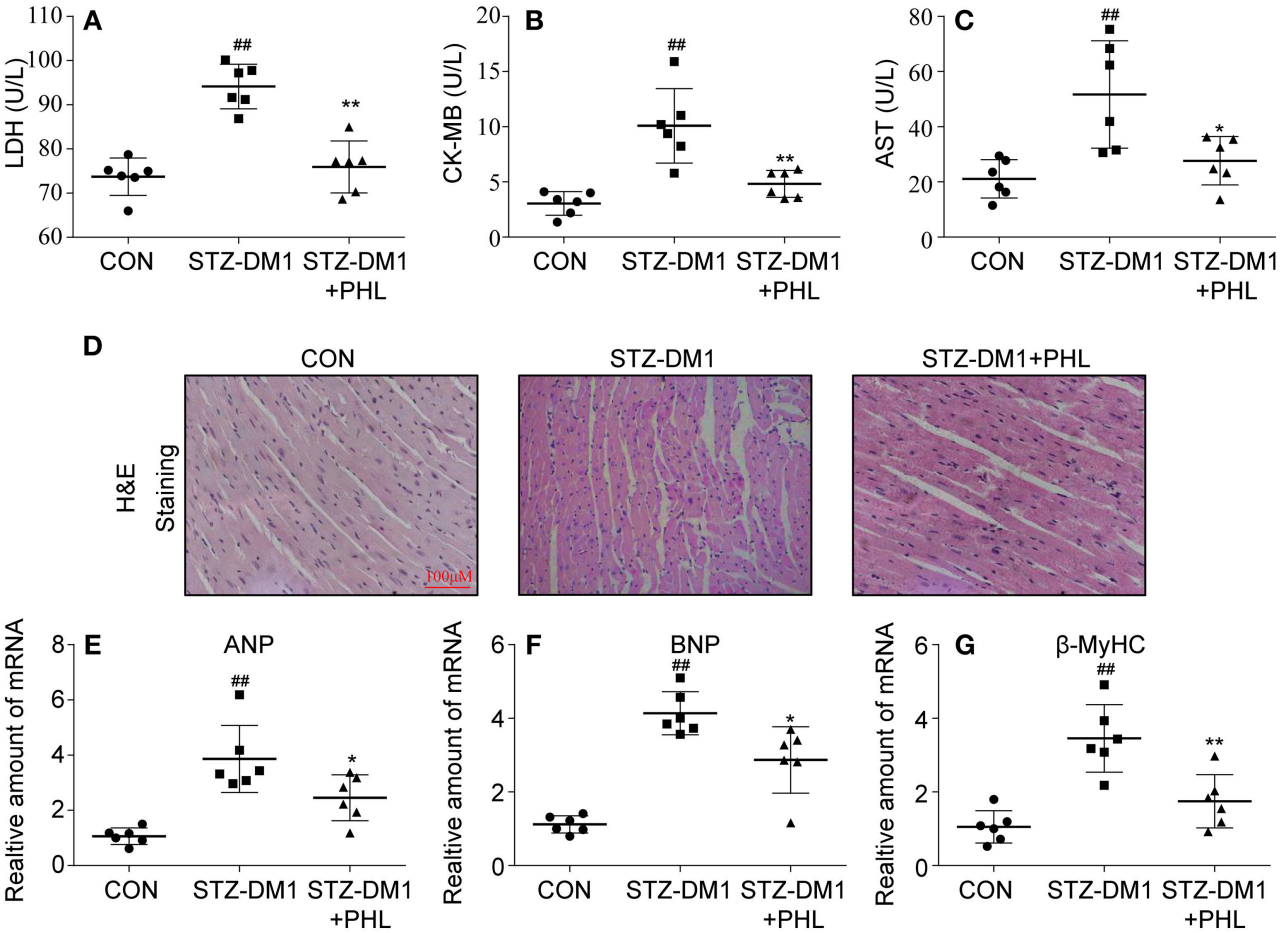
PHL attenuated diabetes-induced cardiac injury. Diabetes mellitus was induced in male C57BL/6 mice by a single intraperitoneal (i.p.) injection of 100 mg/kg STZ and mice with fasting-blood glucose >12 mM were considered diabetes and then diabetic mice were orally treated with phloretin (PHL, 10 mg/kg), or vehicle by gavage once every 2 days for 8 weeks, which was administrated in diabetic mice. Serum levels of LDH **(A)**, CK-MB **(B)**, and AST **(C)** were determined using indicated kits (Six mice in each group were used for above analysis). **(D)** Representative images from H&E sections of heart tissues are shown, × 400 amplification. **(E–G)** The mRNA expression levels of ANP **(E)**, BNP **(F)**, and β-MyHC **(G)** in myocardial tissues of each group were determined by real-time qPCR. Six mice in each group were used for above analysis. ^*^P < 0.05, ^**^P < 0.01 vs. STZ-DM1 group; ^##^*P* < 0.01 vs. CON group.

The *in vitro* experiments clearly showed that PHL could reduce oxidative stress, hypertrophy and fibrosis in H9c2 cells. Next, we evaluated the protected effects of PHL on heart *in vivo*. As shown in Figures 4A–C, PHL significantly inhibited the hyperglycemia-induced upregulation of biochemical markers LDH (Figure 4A), CK-MB (Figure 4B) and AST (Figure 4C) of myocardial injury. The H&E stain revealed that the hyperglycemia-stimulated hearts had structural abnormalities, namely disorganized myofibers. Heart tissues from PHL treatment group showed no significant structural changes compared to the control (CON) group (Figure 4D). Investigation of hypertrophy related genes in heart tissues revealed that PHL significantly attenuated the high expression levels of ANP (Figure 4E), BNP (Figure 4F), and β-MyHC (Figure 4G) found in diabetic mice. However, PHL treatment did not significantly affect fasting weight and blood sugar (Figures S1A,B), suggesting that the cardiac protective effects of PHL were not related to metabolic changes.

### PHL Inhibited Cardiac Oxidative Stress and Fibrosis in the Diabetic Myocardium

**Figure 5:**
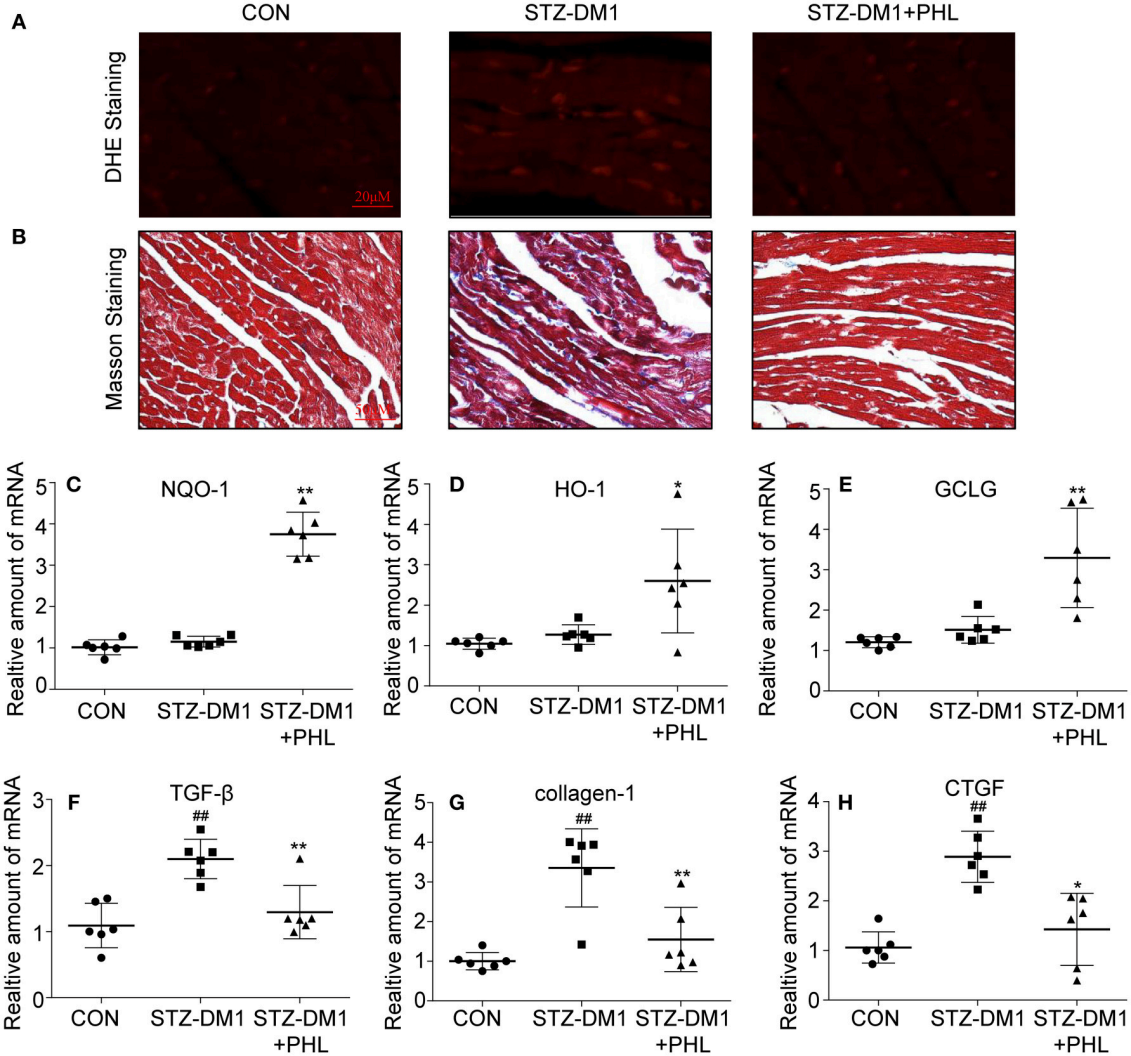
PHL inhibited cardiac oxidative stress and fibrosis in the diabetic myocardium. **(A)** Representative images for DHE staining using the frozen section of heart tissues as described in Materials and Methods (1000 × magnification). **(B)** Assessment of cardiac fibrosis by Masson's Trichrome staining (400 × magnification). **(C–H)** The mRNA expression levels of HO-1 **(C)**, NQO-1 **(D)**, GCLC **(E)**, TGF-β **(F)**, collagen-1 **(G)**, and CTGF **(H)** in myocardial tissues of each group were determined by real-time qPCR. Six mice in each group were used for above analysis. ^*^P < 0.05, ^**^P < 0.01 vs. STZ-DM1 group; ^##^*P* < 0.01 vs. CON group.

To identify potential pharmacological activity for PHL protection *in vivo*, a series of biomarkers related to oxidation and fibrosis were investigated. Firstly, DHE staining showed that PHL attenuated the increase in ROS level in DCM (Figure 5A). Next, connective tissue collagen and histopathology of the hearts from PHL treated and untreated diabetic mice were assessed using Masson's trichrome stain. PHL effectively inhibited the fibrotic process of DCM (Figure 5B). As shown in Figures 5C–E, the expression levels of HO-1, NQO-1, GCLC in DCM tissues were upregulated by PHL. Additionally, in agreement with the histopathology, the high TGF-β (Figure 5F), collagen-1 (Figure 5G), and CTGF (Figure 5H) were significantly downregulated in DCM. These results suggested that the protective effect of PHL on cardiomyocyte in hyperglycemia-induced DCM might be related to its anti-oxidant and anti-fibrotic function.

### PHL Targeted Keap1 Leading to Dissociation of the Keap1/Nrf2 Complex

Keap1/Nrf2 signaling pathway play a major role in regulating oxidative stress (28). Increasing evidence showed that Keap1/Nrf2 signaling pathway regulates inflammation, fibrosis and endoplasmic reticulum stress (ER stress) in DCM (29, 30). The *in vitro* study revealed that PHL promoted Nrf2 expression in hyperglycemia stimulation but the target and mechanism remained unclear. Previous studies indicated that Nrf2 maintains an inactive state in the cytoplasm under unstressed conditions bound to its inhibitor Keap1 (29), a vital regulator of the anti-oxidant response. Dissociation of the protein-protein interaction between Nrf2 and Keap1 leads to expression of detoxifying anti-oxidant enzymes. Thus, molecular modeling and immunoblotting analysis were applied, focusing particularly on the hydrophobic binding site of Keap1.

**Figure 6:**
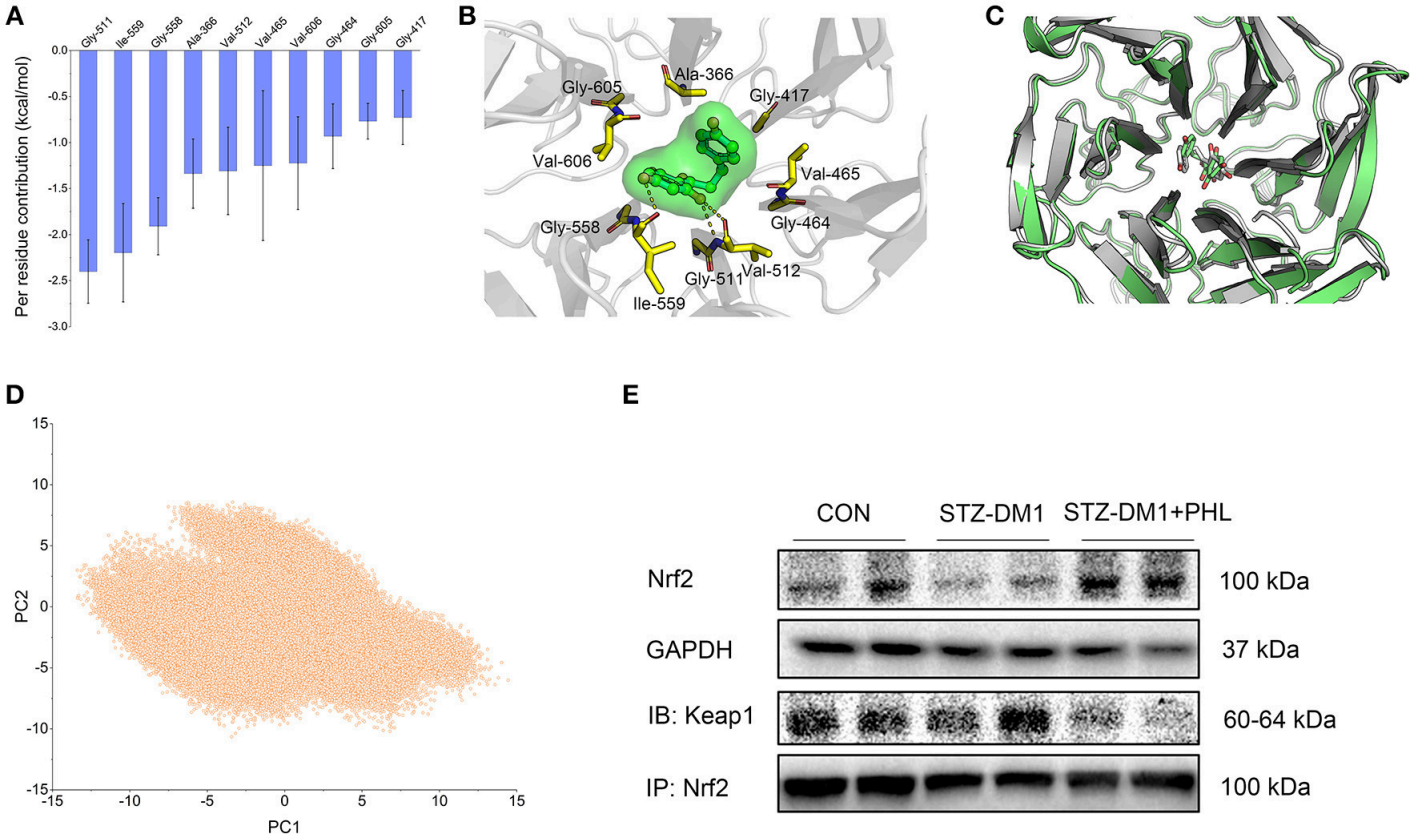
PHL targeted Keap1 to disassemble the Keap1/Nrf2 complex. **(A)** Per-residue of top 10 contribution to the binding free energy; **(B)** Structural analysis of the most 10 contributors of Keap1 to PHL, hydrogen bonds are colored yellow; **(C)** Alignment of the representative structures between from conventional MD simulation and from GaMD simulation; **(D)** PCA scatter plot of 200,000 snapshots from GaMD simulation along the first two principal components. **(E)** Immunoblotting analysis of Nrf2 expression and Co-Immunoprecipitation analysis of Nrf2 and Keap1 complex in lysates prepared from heart tissues. GAPDH was used as a loading control.

Molecular docking was used to generate the initial structure and molecular dynamics (MD) simulations were applied to investigate the dynamic behavior. In order to monitor the stability of Keap1/phloretin complex during MD simulation, the root-mean-square deviations (RMSDs) of the backbone atoms (C_α_) of Keap1 and the heavy atoms of PHL were analyzed (Figure S2). As shown in Figures S2A,B, the RMSD values of the backbone atoms of Keap1 and the heavy atoms of phloretin have a small fluctuation during the whole conventional MD simulation. GaMD simulation indicated that the Keap1/PHL complex was relatively stable. To gain an insight into the roles of individual residues in determining the interaction between Keap1 and PHL, the binding free energy decomposition was carried out. As shown in Figure 6A, the 10 most contributed residues were Gly-511, Ile-559, Gly-558, Ala-366, Val-512, Val-465, Val-606, Gly-464, Gly-605, and Gly-417. The predominant interactions were hydrogen bonds (residues of Ile-559, Gly-511, and Val-512) and hydrophobic interactions (Figures 6A,B).

Due to possible energy barriers between various intermediate states, conventional MD simulations cannot sample the conformational ensembles. Hence, an enhanced sampling technique to speed up the conformational sampling and take conformational samples at various intermediate states is needed. The traditional enhanced sampling methods often require predefined reaction coordinates (RCs), such as RMSD, atom distances, eigenvectors generated from the principal component analysis, which usually requires rich experience of the simulated systems. Nevertheless, the enhanced sampling method of GaMD simulation avoids such a requirement. Compared with conventional MD simulation, GaMD simulation can take samples at various intermediate states by adding a harmonic boost potential to smoothen the system potential energy surface, which are not accessible to conventional MD simulations. Therefore, the enhanced sampling method, GaMD simulation, was carried out to enhance conformational sampling (19, 20). Alignment of the representative structures from conventional MD and GaMD simulations exhibited high similarity with minor adjustments, indicating that PHL in the binding site of Keap1 was sufficiently stable (Figure 6C). The principal component analysis (PCA) further supported this observation (Figure 6D). Theoretically, when principal components are plotted against each other, similar structures are clustered and each cluster shows a different protein conformational state. As shown in Figure 6D, only one cluster was observed, the target for PHL is likely to be Keap1. To verify this theory, the expressions of Keap1/Nrf2 complex in the heart tissues were assessed that the expressions level of Nrf2 in diabetic heart tissues. The results showed that the expression of Nrf2 was significantly lower and the levels of Keap1/Nrf2 complex was a little higher in diabetic heart tissues, while PHL inhibited this process (Figure 6E and Figure S4). In summary, the computational and experiment results demonstrated that Keap1 may be the direct target for PHL to exert anti-oxidative effects and promoted Nrf2 expression for the protective effects in DCM.

## Discussion

It has been well-established that persistent hyperglycemia in diabetic patients induce cardiomyocyte hypertrophy, myocardial inflammation, fibrosis, and apoptosis (1, 2, 5, 8). As observed in our study, high glucose induced oxidative stress, hypertrophy, and fibrosis in myocardial cells and diabetic mice (Figures 2–5). Current therapies for DCM focus on intensive blood glucose control (31). However, this strategy does not prevent the development of cardiac complications associated with hyperglycemia. Treatment with anti-diabetics, such as miglitol, significantly reduced the blood glucose via different mechanisms *in vivo*, while the diabetic complications remained (32). Moreover, a few studies showed that anti-inflammatory agents prevented the development of cardiac and renal injury in streptozotocin (STZ)-induced diabetic mice (2). Meanwhile, a large number of targets, such as TLR4 and FGFR as well as fatty acid-induced cardiac remodeling have been investigated (33, 34). Therefore, we speculate that there are unknown mechanisms contributing to cardiac fibrosis, cardiac remodeling, and cell death.

Recently, natural products have been increasingly evaluated for their effects on cardiovascular diseases. Most notably, flavonoids exhibited properties of inflammation, oxidant stress, cell death, and fibrosis by direct or indirect mechanisms (35, 36). It was recently been reported that several flavonoids, such as quercetin, kaempferol, liquiritin, and baicalein exhibited multiple effects in diabetic complications by inhibiting inflammation, oxidation and fibrosis (37–40). PHL, a natural flavonoid compound derived from apples and pears, was shown to reduce myocardial hypertrophy in acute models of heart disease (41). In addition, PHL prevents high-fat diet-induced obesity and improves metabolic homeostasis through anti-oxidative effect (42). However, to-date, no data is available regarding the effects of PHL on DCM. In this study, we investigated the activities and mechanisms of PHL in an *in vivo* model for DCM. Following STZ injection for 10 weeks, cardiac remodeling including hypertrophy, cardiomyocyte disorganization, and fibrosis were observed in the hearts of diabetic mice. Interestingly, oral PHL (10 mg/kg) attenuated cardiac remodeling without any effects on the glucose level or body weight of diabetic mice (Figure S1, Figures 2–5). Similar biochemical results were reflected in hyperglycemia-treated H9c2 cells (Figures 2, 3). It is interesting that PHL attenuated MDA level and upregulated Nrf2, HO-1, and NQO-1 expressions *in vitro* (Figure 2). These findings suggested that PHL has a potential therapeutic effect in DCM, likely through decreased cardiac oxidative stress.

Increasing evidence has shown that oxidative stress plays an important role in the pathophysiology of hyperglycemic induction of cardiovascular disease (43, 44). Cardiac oxidative stress is associated with increased cardiac fibrosis and cell death, leading to the development of severe heart failure (43, 44). Excess oxidation results in ROS aggregation in tissues. In metabolic disease, sustained production of ROS is critical to the development of cardiac injuries. ROS induced mitochondrial DNA has been proposed to be particularly susceptible to oxidative damage. In this study, PHL significantly reduced hyperglycemia-induced ROS accumulation in H9c2 cells and heart tissues from diabetic mice (Figure 2A and Figure 5A). There is an increasing recognition that Keap1/Nrf2 pathway activation can be beneficial for DCM, as it has been recognized as a key regulator of anti-oxidant defense system by mediating the expression of anti-oxidant genes, such as HO-1, GCLC, NAPD-H, and NQO-1. While hyperglycemia reduced the expression of Nrf2, HO-1, and NQO-1, PHL markedly upregulated these genes *in vitro* and *in vivo* (Figure 2, Figure 5). Interestingly, Keap1 was increased by hyperglycemia, but PHL downregulated Keap1 protein levels. Using molecular modeling and immunoblotting, PHL was determined to bind to Keap1 via hydrogen bonds and hydrophobic interactions (Figures 6A–D), which subsequently resulted in increased available Nrf2.

In conclusion, this study demonstrated that PHL could prevent the hyperglycemia-induced cardiac injury. The possible mechanism involved, in prevention of oxidative stress and related cytoprotective effect, could be through degradation of Keap1 and upregulation of Nrf2 expressions, leading to downstream regulation of key detoxifying enzymes (Figures 2–6 and Figure S3). Thus, PHL could be a candidate for treating diabetic complications, especially for DCM. In addition, conventional MD and GaMD simulations (Figures 6A–D) supported the hypothesized mechanism whereby PHL protected cardiomyocyte from hyperglycemia through disruption of the interaction between Keap1 and Nrf2. Co-IP and western blot analyses also supported this hypothesis (Figure 6E and Figure S4). However, it is still not clear whether PHL induced Nrf2 transcription and led to elimination of ROS or whether PHL inhibited Keap1/Nrf2 complex first and the subsequent antioxidant response. And the mechanism of PHL how to induced Nrf2 transcription is unknown. The approach using shRNA knockdown *in vitro* with hyperglycemia would be more robust to solve these shortages. This is a limitation of this study.

Overall, this study strongly supports PHL as a promising natural agent via increased Nrf2 expression dissociation of Keap1/Nrf2 complex, leading to decreased cardiac oxidative stress in DCM. At the same time, this study also highlighted Keap1/Nrf2 pathway as a potential therapeutic target for DCM management.

## Author Contributions

YY and JJ who originally designed the project, performed the research, analyzed data, and wrote the manuscript draft. PS, HW, and LY performed partial experiments and data collection. XW were responsible for revising the manuscript. All authors approved the final version of the manuscript.

### Conflict of Interest Statement

The authors declare that the research was conducted in the absence of any commercial or financial relationships that could be construed as a potential conflict of interest.
