# Supplementary material for: Phloretin Prevents Diabetic Cardiomyopathy by Dissociating Keap1/Nrf2 Complex and Inhibiting Oxidative Stress
Source: Front Endocrinol (Lausanne). 2018 Dec 20;9:774. doi: 10.3389/fendo.2018.00774 (PMC6306411; doi:10.3389/fendo.2018.00774)
Supplement: Supplementary file 1 [file Data_Sheet_1.docx]

**Supporting information**

**Phloretin Prevents Diabetic Cardiomyopathy by Dissociating Keap1/Nrf2 Complex and Inhibiting Oxidative Stress**

Yin Ying^1, #^, Jiye Jin^2, #^, Li Ye^3^, Pingping Sun^1^, Hui Wang^1^, Xiaodong Wang^4,^ *

^1^ Department of Pharmacy, Tongde Hospital of Zhejiang Province, Hangzhou 310012, Zhejiang, China.

^2^ Department of Rehabilitation, Tongde Hospital of Zhejiang Province, Hangzhou 310012, Zhejiang, China.

^3^ Department of Nursing, Tongde Hospital of Zhejiang Province, Hangzhou 310012, Zhejiang, China.

^4^ Department of Vascular Surgery, Tongde Hospital of Zhejiang Province, Hangzhou 310012, Zhejiang, China.

^#^ These authors contributed equally to this work

***Corresponding author:**

Xiaodong Wang

Department of Vascular Surgery, Tongde Hospital of Zhejiang Province, 234 Gucui Road, Hangzhou 310012, Zhejiang, China.

E-mail: wangxiaodong155@gmail.com; Phone: 86-571-89972000

**Table S1. Primer sequences of genes in RT-qPCR assay**

| **Gene** | **Species** | **Forward Primer** | **Reversed Primer** |
| --- | --- | --- | --- |
| NQO-1 | Mouse | TTCTGTGGCTTCCAGGTCTT | AGGCTGCTTGGAGCAAAATA |
| collagen-1 | Mouse | TGGCCTTGGAGGAAACTTTG | CTTGGAAACCTTGTGGACCAG |
| TGF-β | Mouse | TGACGTCACTGGAGTTGTACGG | GGTTCATGTCATGGATGGTGC |
| ANP | Mouse | AACCTGCTAGACCACCTGGA | TGCTTTTCAAGAGGGCAGAT |
| β-actin | Mouse | CCGTGAAAAGATGACCCAGA | TACGACCAGAGGCATACAG |
| HO-1 | Mouse | AAGAGGCTAAGACCGAATTC | GCATAAATTCCCACTGCCAC |
| CTGF | Mouse | ACTATGATGCGAGCCAACTGC | TGTCCGGATGCACTTTTTGC |
| GCLC | Mouse | GCCCTACGGAGGAACGATG | CTAGTCTGGGGAATGAAGTGATG |
| BNP | Mouse | GTCAGTCGTTTGGGCTGTAAC | AGACCCAGGCAGAGTCAGAA |
| β-MyHC | Mouse | GCCAAGACTGTCCGGAATGA | TGGAAGATCACCCGGGACTT |
| NQO-1  collagen-1  HO-1  TGF-β  ANP  β-actin | Rat  Rat  Rat  Rat  Rat  Rat | ACCTTGCTTTCCATCACCAC  GAGCGGAGAGTACTGGATCGA  TCTATCGTGCTCGCATGAAC  GGACTACTA CGCCAAAGAAG  CTGCTAGACCACCTGGAGGA  ATCGTGGGCCGCCCTAGGCACC | CAAAGGCGAAAACTGAAAGC  CTGACCTGTCTCCATGTTGCA  CAGCTCCTCAAACAGCTCAA  TCAAAAGACAGCCACTCAGG  AAGCTGTTGCAGCCTAGTCC  CTCTTTAATGTCACGCACGATTTC |
| CTGF | Rat | GCCTGTTCCAAGACCTGT | GGATGCACTTTTTGCCCTTCTTA |
| GCLC | Rat | TGGCCACTATCTGCCCAATT | CCCCAGCAATCAATGTC |
| BNP | Rat | GATCCAGGAGAGACTTCGAAA | CGGTCTATCTTCTGCCCAA |
| Nrf2 | Rat | ACTGTCCCCAGCCCAGAGGC | CCAGGCGGTGGGTCTCCGTA |
| β-MyHC | Rat | GAGGAGAGGGCGGACATT | ACTCTTCATTCAGGCCCTTG |


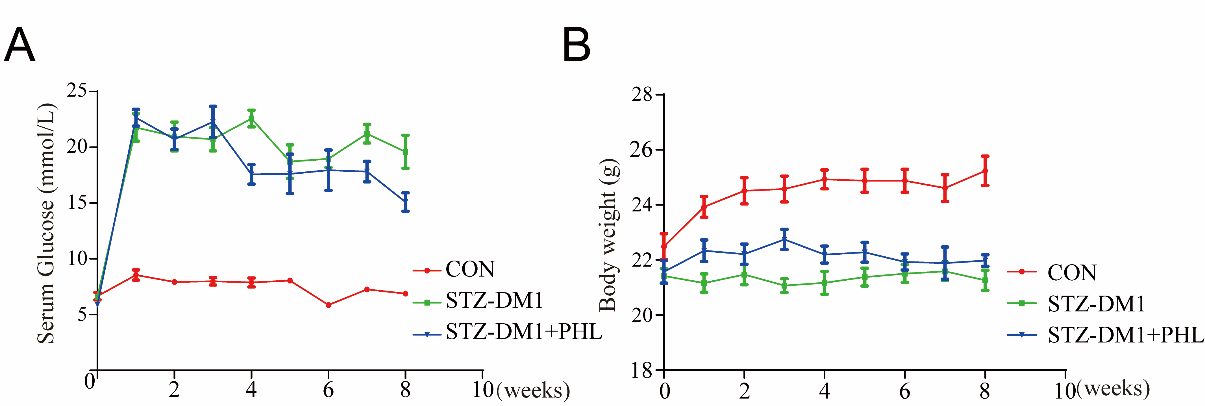


**Figure S1.** Serum glucose (A) and body weight (B) of STZ-DM1 mice models. (n=8 each group.)

**
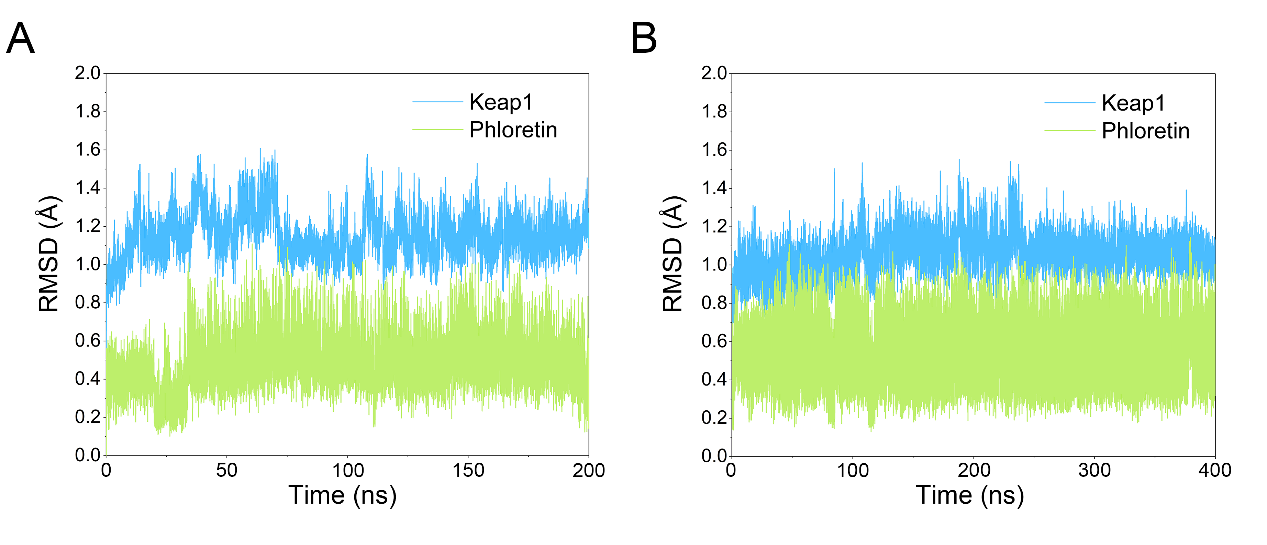
**

**Figure S2.** Time evolution of the root-mean-square deviation (RMSD) of Keap1 and phloretin from conventional MD simulation (A), and from GaMD simulation (B).

**
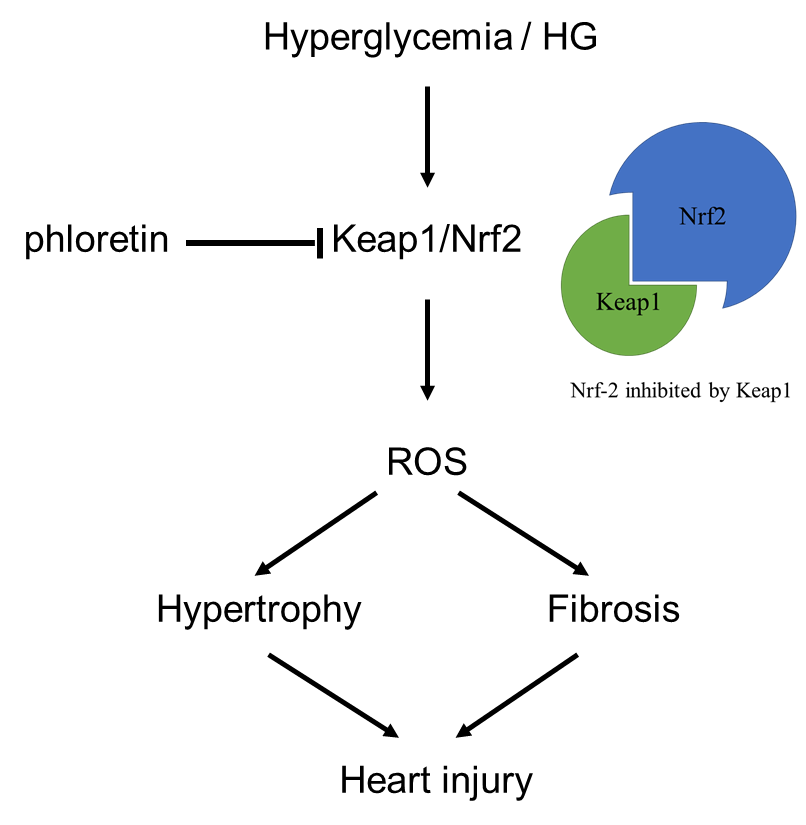
**

**Figure S3.** The mechanism of the protection provided by phloretin, via targeting Keap1/Nrf2 axis, against hyperglycemia-induced diabetic cardiac injury.


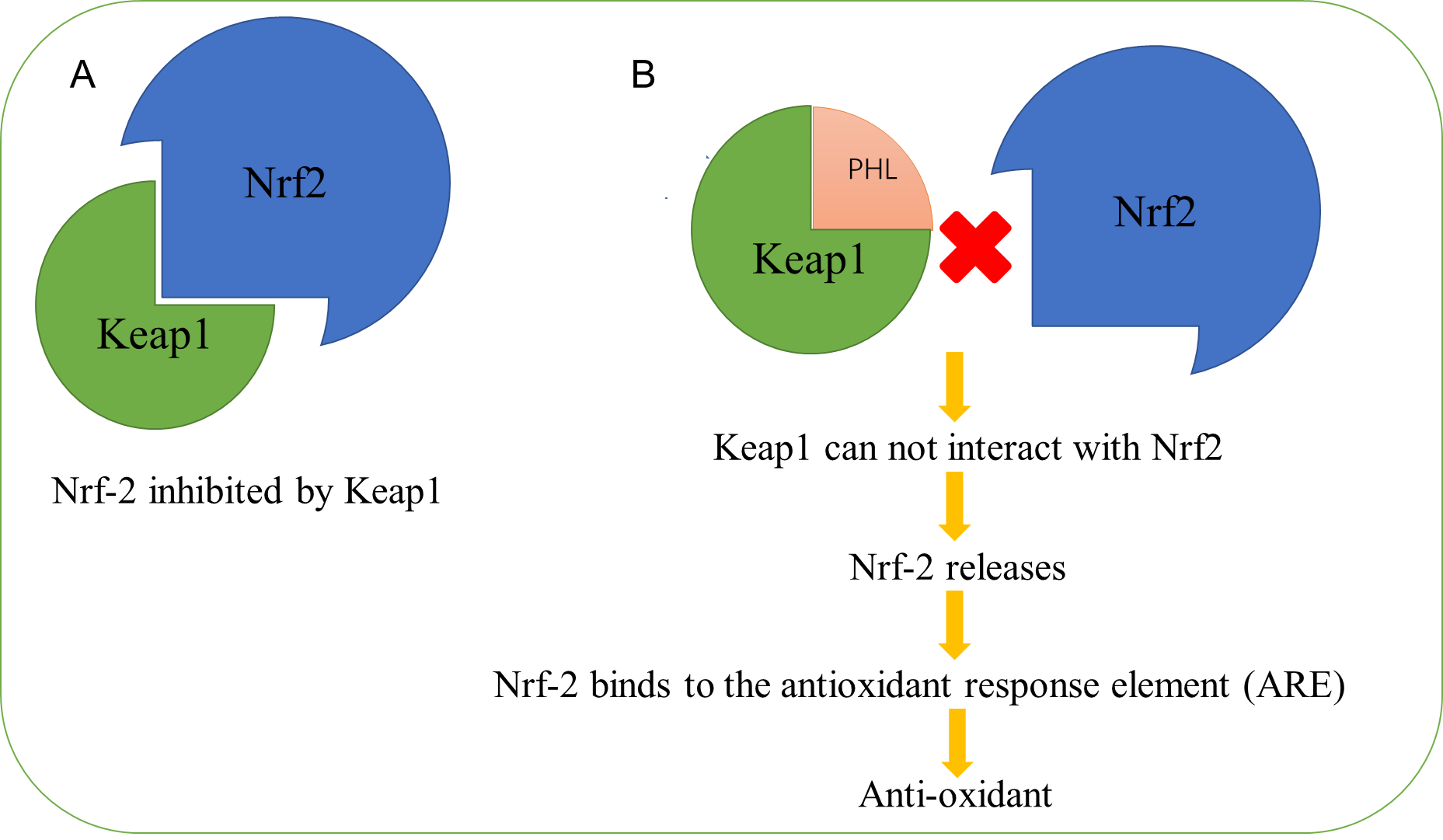


**Figure S4.** The diagrammatic drawing of Nrf2/Keap1 (A) and PHL/Nrf2/Keap1 (B).
